# Supplementary material for: A Compositional Model to Predict the Aggregated Isotope Distribution for Average DNA and RNA Oligonucleotides
Source: Metabolites. 2021 Jun 18;11(6):400. doi: 10.3390/metabo11060400 (PMC8234063; doi:10.3390/metabo11060400)
Supplement: Supplementary file 1 [file metabolites-11-00400-s001.zip › Supplementary materials_Valkenborg.pdf]

A compositional model to predict the aggregated isotope distribution for average DNA and RNA oligonucleotides.

Agten, Annelies*[†,‡]; Prostko, Piotr*[†,‡]; Geubbelmans, Melvin[†,‡]; Liu, Youzhong[§]; De Vijlder, Thomas[§]; Valkenborg, Dirk[†,‡]

*both authors contributed equally

[†] Data Science Institute, Hasselt University, Agoralaan 1, BE 3590 Diepenbeek, Belgium

[‡] Interuniversity Institute for Biostatistics and Statistical Bioinformatics (I-BioStat), Agoralaan 1, BE 3590 Diepenbeek, Belgium

[§]Chemical & Pharmaceutical Development & Supply, Janssen Research & Development, Turnhoutseweg 30, BE2340 Beerse, Belgium

Table of Contents

Supplementary materials DNA and RNA database generation 2

Supplementary materials DNA model 3

Supplementary materials RNA model 5

Supplementary materials for compound: DNA_short1. 7

Supplementary materials for compound: DNA_SHORT2. 9

Supplementary materials for compound: RNA-like 12

Supplementary materials for the software 14

Supplementary materials DNA and RNA database generation

Table S.1: Basis components of nucleotides: DNA/RNA nucleobases, sugar structures, phosphate . The table contains the names used in this manuscript and the elemental composition.

	C	H	N	O	S	P
Adenine	5	5	5	0	0	0
Cytosine	4	5	3	1	0	0
Guanine	5	5	5	1	0	0
Thymine	5	6	2	2	0	0
Uracil	4	4	2	2	0	0
Ribose	5	10	0	5	0	0
Deoxyribose	5	10	0	4	0	0
Phosphate	0	3	0	4	0	1

Table S.2: Nucleotides, abbreviation and construction of the nucleotides.

Nucleotides	Construction
dAMP	adenine+deoxyribose-H2O+phosphate-H2O
dCMP	cytosine+deoxyribose-H2O+phosphate-H2O
dGMP	guanine+deoxyribose-H2O+phosphate-H2O
dTMP	thymine+deoxyribose-H2O+phosphate-H2O
AMP	adenine+ribose-H2O+phosphate-H2O
CMP	cytosine+ribose-H2O+phosphate-H2O
GMP	guanine+ribose-H2O+phosphate-H2O
UMP	uracil+ribose-H2O+phosphate-H2O

Supplementary materials DNA model

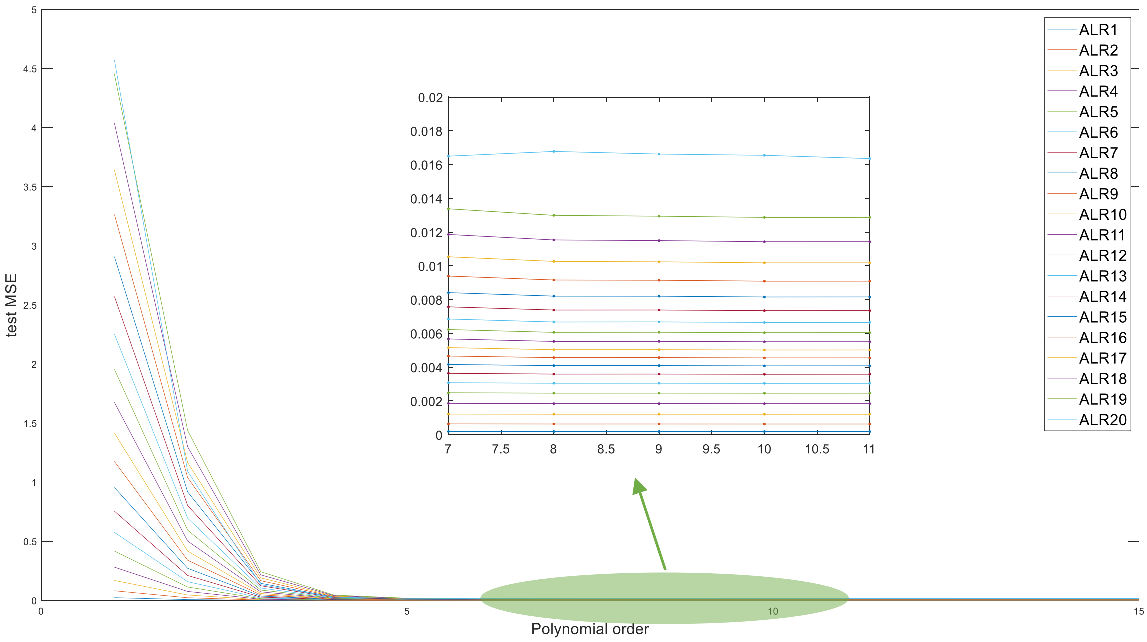

Figure S.1: Evolution of the test mean squared error (MSE) in function of the polynomial order. Each line represents the model fit of an ALR transformed DNA isotope.

Table S.3: Test MSE of the separate model fits on the 20 ALR-transformed DNA isotopes for order of the polynomial from 8 to 11. The minimum test MSE is highlighted in grey.

		ORDER OF THE POLYNOMIAL MODEL			
		8	9	10	11
ALR-TRANFORMED ISOTOPES	1	0.000186397	0.000186747	0.000186301	0.000186287
	2	0.000633685	0.000633941	0.000632897	0.000633063
	3	0.001214316	0.001214537	0.001212473	0.001212958
	4	0.001841652	0.001842098	0.001839374	0.001839865
	5	0.002462248	0.002463168	0.002459398	0.002460283
	6	0.003048585	0.003049996	0.003042783	0.003044823
	7	0.003590845	0.003592439	0.003579840	0.003582810
	8	0.004092115	0.004093534	0.004076269	0.004079117
	9	0.004566120	0.004567293	0.004547883	0.004549982
	10	0.005034696	0.005036136	0.005016409	0.005017999
	11	0.005524371	0.005526958	0.005506387	0.005508003
	12	0.006062695	0.006066736	0.006042770	0.006044672
	13	0.006675238	0.006679524	0.006649235	0.006651324
	14	0.007384007	0.007385721	0.007347318	0.007349344
	15	0.008207564	0.008203087	0.008156395	0.008158173
	16	0.009162473	0.009148490	0.009094473	0.009095941
	17	0.010265304	0.010239638	0.010179520	0.010180662
	18	0.011534488	0.011496457	0.011430950	0.011431679
	19	0.012991604	0.012941995	0.012870871	0.012870950
	20	0.016777407	0.016621944	0.016547568	0.016354290

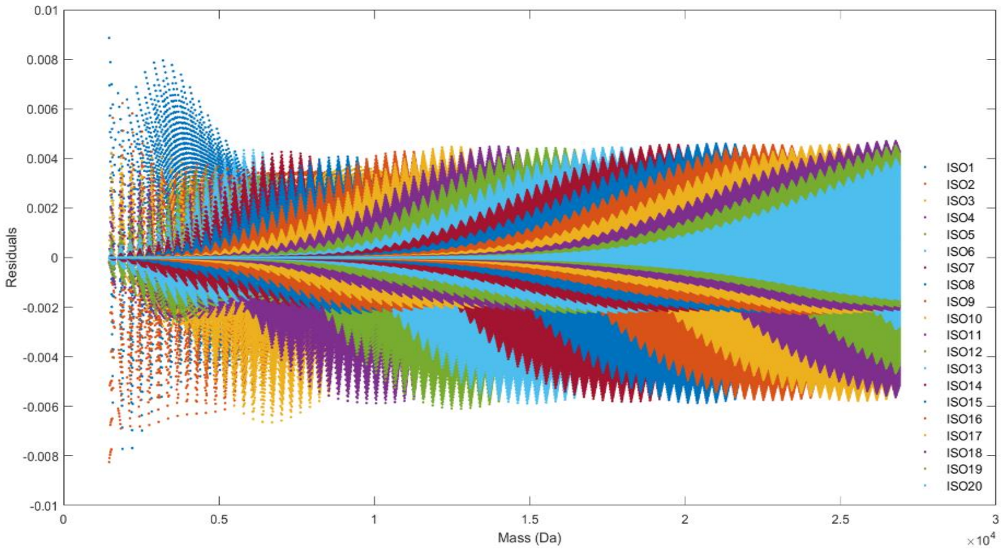

Figure S.2: Overlay plot of the probability residuals for the first 20 DNA isotopes. The different isotope residuals are colour-coded and follow a trend in relation to the mass. The y-axis denotes the difference between the theoretical and predicted isotope probabilities. It can be observed that the majority of the residuals fall within an error of 0.4% and -0.6%.

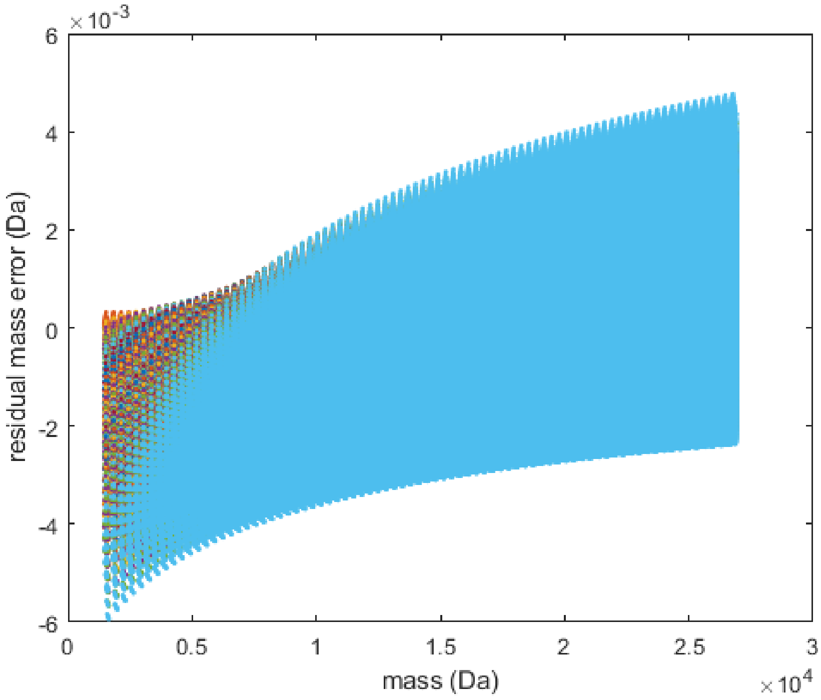

Figure S.3: Overlay plot of the mass residuals for the DNA model. The different isotope mass residuals are colour-coded and follow a trend in relation to the mass. The y-axis denotes the difference between the theoretical centroid mass of an aggregated isotope variant and the monoisotopic variant with the difference predicted by the average mass model.

Supplementary materials RNA model

Table S.4: Standardisation values for the monoisotopic mass covariate of the RNA database.

Mu	23151.5158
Sigma	4883.1422

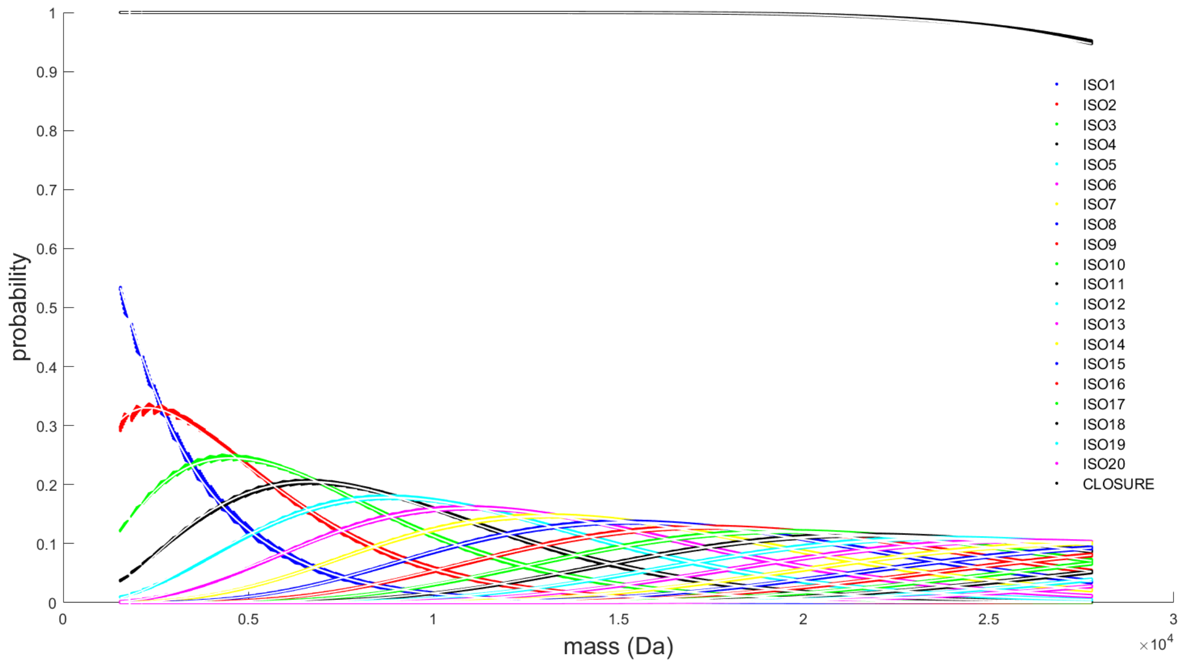

Figure S.4: Scatterplot of the first 20 isotopes of all possible RNA molecules within the restricted mass range between 1543.2170 Da and 27776.7667 Da. Every isotope variant is denoted by a different colour coding. The plot illustrates how the probability (y-axis) for a particular aggregated isotope variant evolves in function of the monoisotopic mass (x-axis). The black line on the top of the figure is the coverage that sums the probabilities of the first 20 isotopes per RNA molecule.

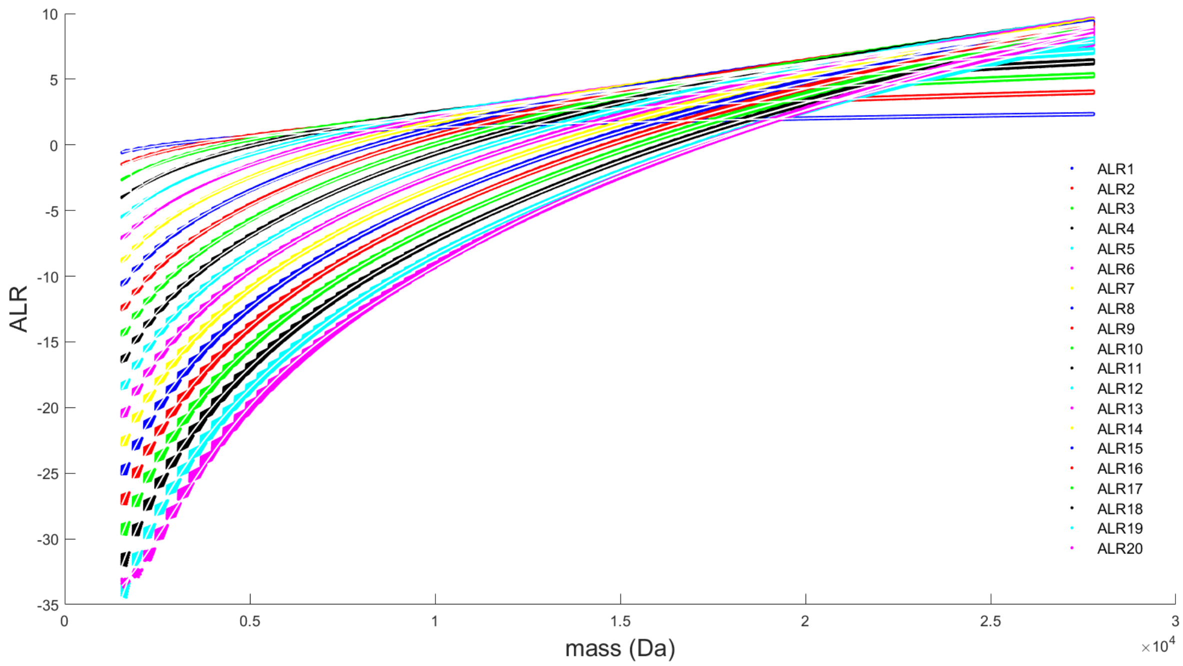

Figure S.5: Scatterplot of the ALR RNA isotopes ratios. The monoisotopic variant is taken as the reference isotope for this transformation. ALR20 is the additive log ratio transformation of the pseudo-isotope that is derived from the coverage term in Figure S.4.

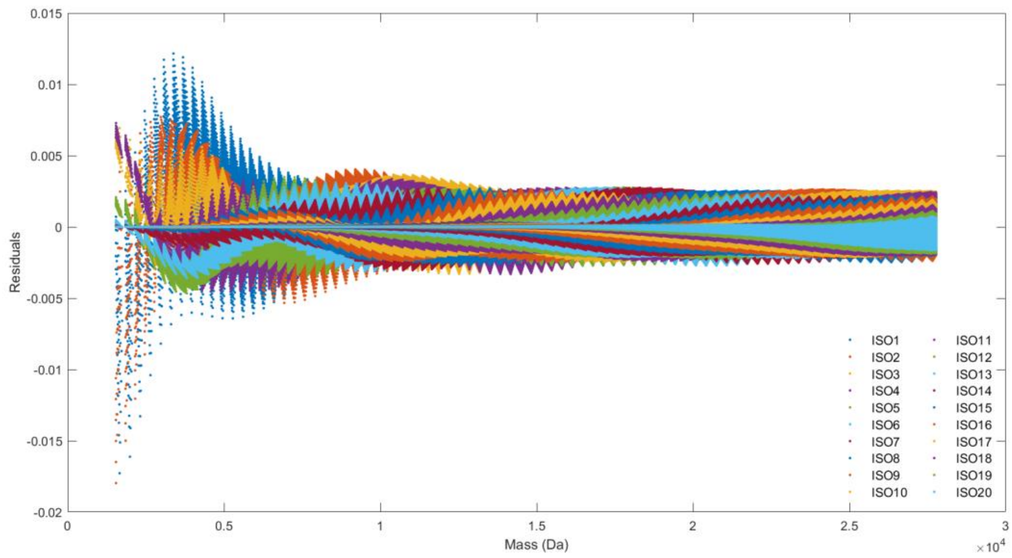

Figure S.6: Overlay plot of the probability residuals for the first 20 RNA isotopes. The different isotope residuals are colour-coded and follow a trend in relation to the mass. The y-axis denotes the difference between the theoretical and predicted isotope probabilities. It can be observed that the majority of the residuals fall within an error of 0.25% and -0.25%.

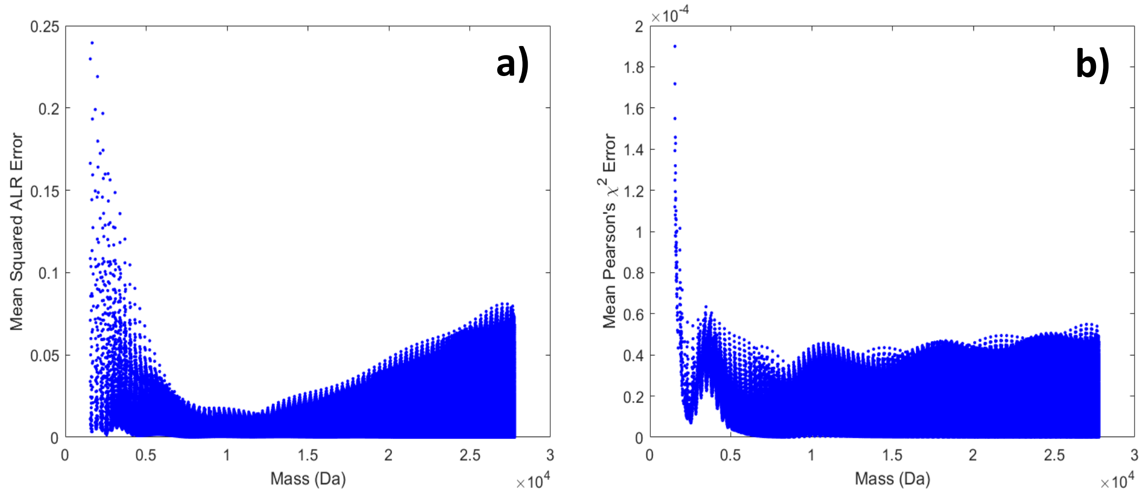

Figure S.7: Panel a) provides the mean squared error in ALR space for the RNA molecules in the restricted mass range. For each of the first 20 isotopes the squared error is computed between the theoretical ALR transformed isotope and the predicted ALR isotope from the final model. Next, the mean squared error for every RNA molecule is computed by taking the mean of the error over the first 20 isotopes. Panel b) provides a similar graphic, except the error is computed as Pearson's chi-squared error in simplex (i.e. probabilities) space.

Supplementary materials for compound: DNA_short1.

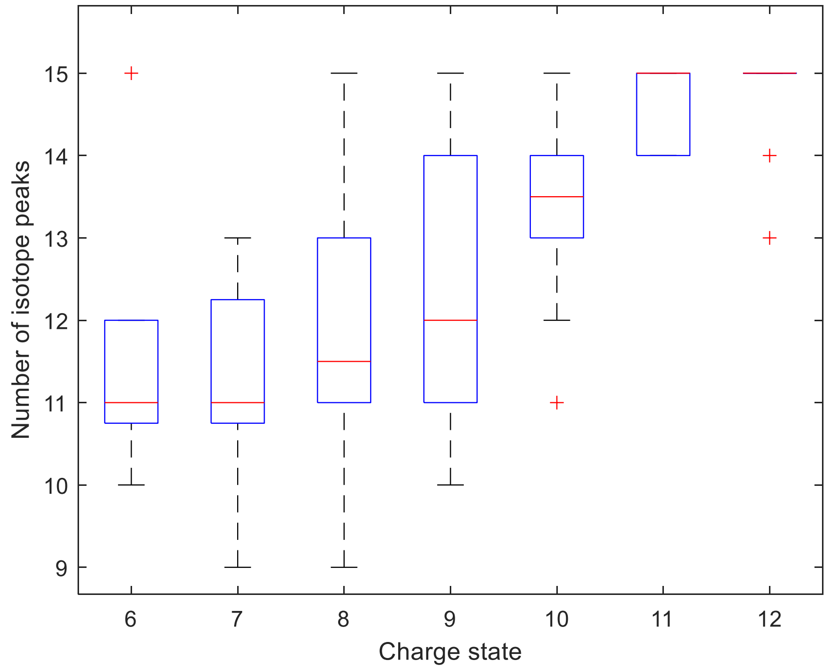

Figure S.8: Boxplots indicating the distribution of the number of observed isotope peaks in relation to the charge state. Each box composes 10 repeated measurements of the DNA_short1 compound over the LC-dimension of the experiment.

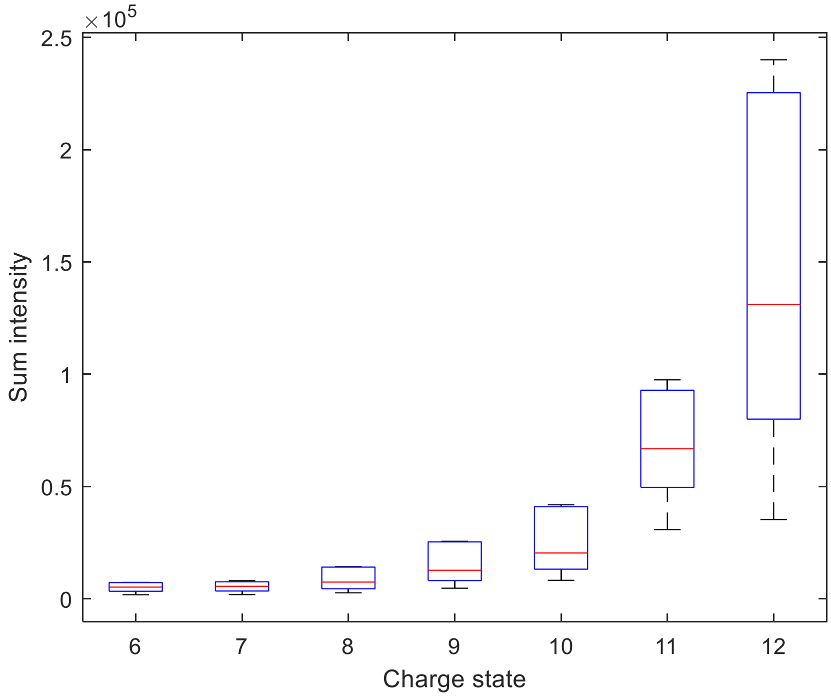

Figure S.9: Boxplots indicating the distribution of the AUC or sum intensity of an observed isotope pattern. Each box composes 10 repeated measurements of the DNA_short1 compound over the LC-dimension of the experiment.

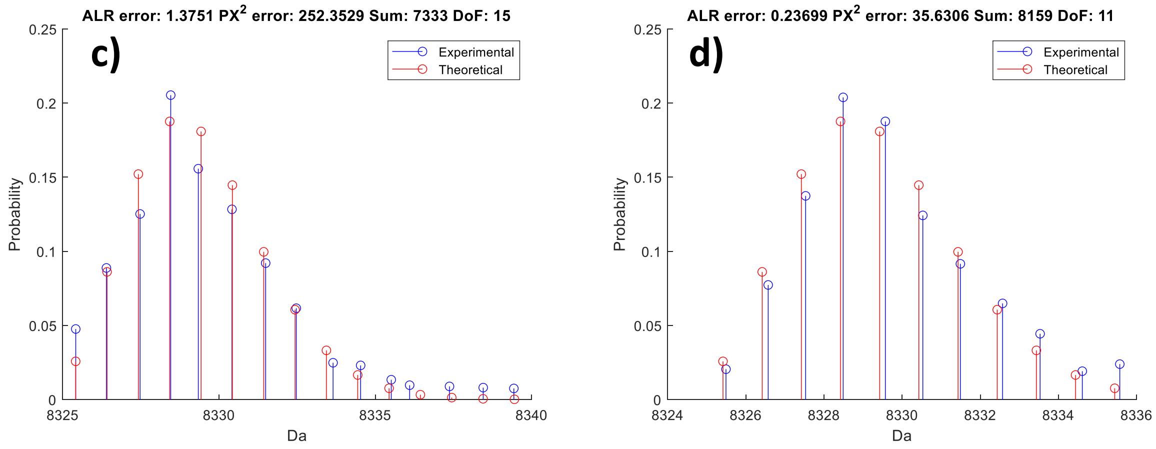

Figure S.10: Stem plot illustrating the observed isotope distribution (blue) and theoretical isotope distribution computed by BRAIN using the elemental composition (red). The red lines are the same for both panels. Panel c) is case c) in Figure 6, whilst panel d) is case d). An important remark should be made here with respect to the scaling. In order to keep the y-axis comparable across different intensity values, we transform the observed intensities to probabilities. Since the identity of the compound is known we can also compute/predict the theoretical/predicted probabilities and sum these for the observed aggregated isotope variants. Next, the intensities will be scaled to that sum probability. In a sense this calculation is the reciprocal of the operation specified in Equation 10.

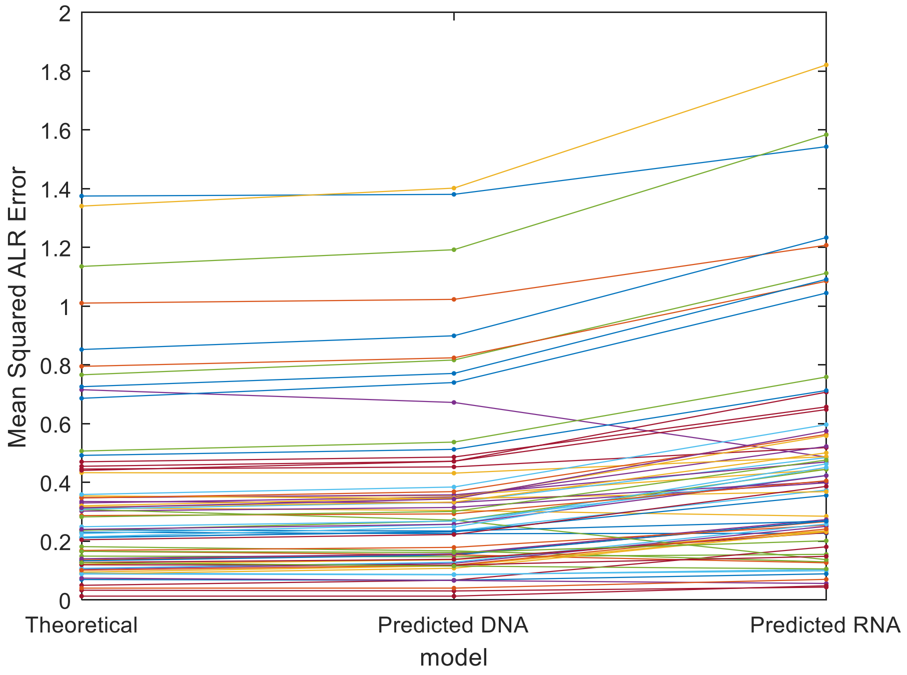

Figure S.11: Spaghetti plot of the Mean Squared ALR error of each of the 68 investigated spectra of the DNA_short1 compound. The dots give the MSE in ALR space for the theoretical, DNA prediction and RNA prediction model. The lines between the errors from the theoretical and DNA prediction model are near horizontal, indicating a good fit. The lines between the error for the DNA and RNA prediction model illustrate a clear incline, indicating that model misspecification has an influence.

Supplementary materials for compound: DNA_SHORT2.

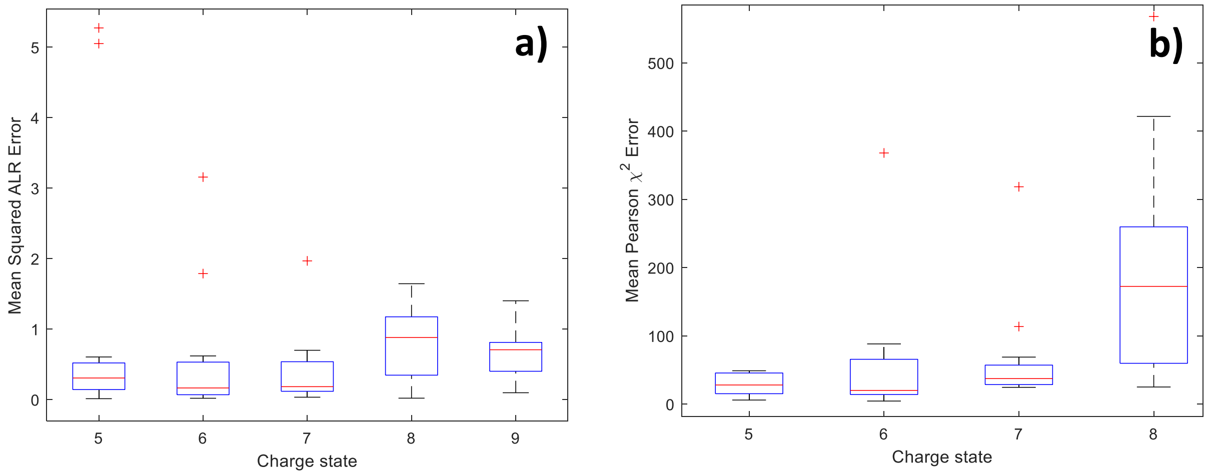

Figure S.12: The error distributions for compound DNA_SHORT2 are provided as Tukey's box and whisker plots across the different charge states. Each box composes 14 repeated measurements of the compound over the LC-dimension of the experiment. Panel a) gives the distribution of mean squared ALR error. Panel b) gives the distribution of the mean Pearson's chi-squared error.

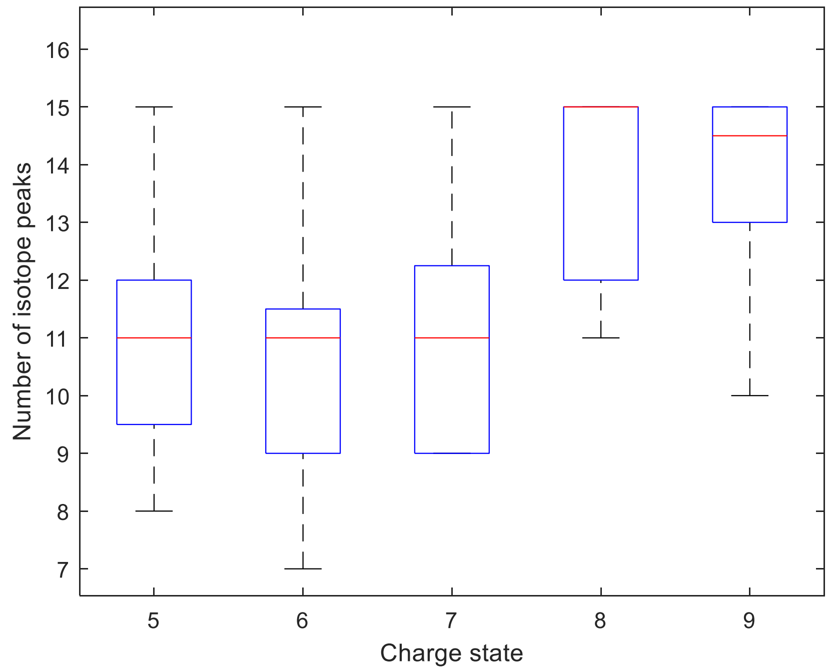

Figure S.13: Boxplots indicating the distribution of the number of observed isotope peaks in relation to the charge state. Each box composes 14 repeated measurements of the DNA_SHORT2 compound over the LC-dimension of the experiment.

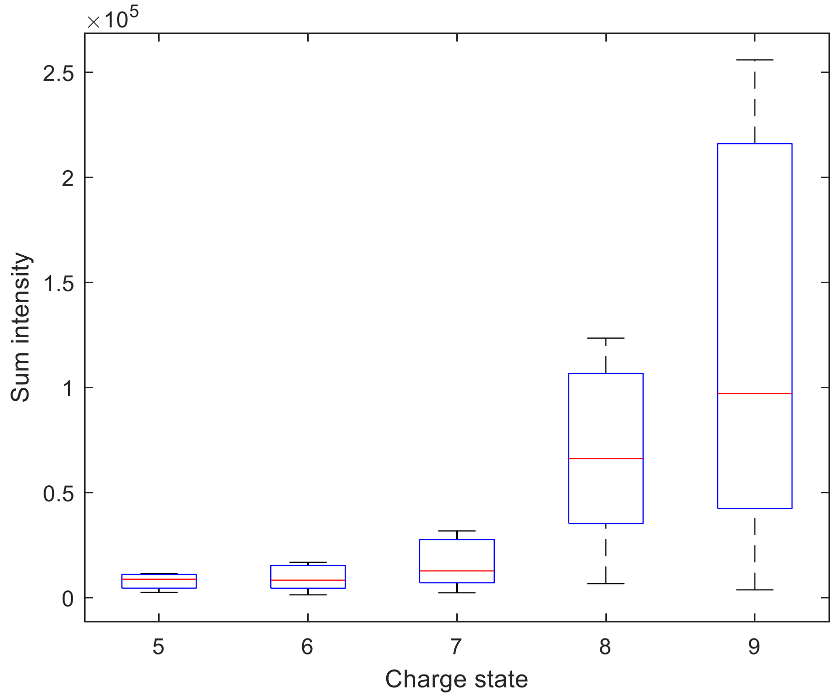

Figure S.14: Boxplots indicating the distribution of the AUC or sum intensity of an observed isotope pattern. Each box composes 14 repeated measurements of the DNA_SHORT2 compound over the LC-dimension of the experiment.

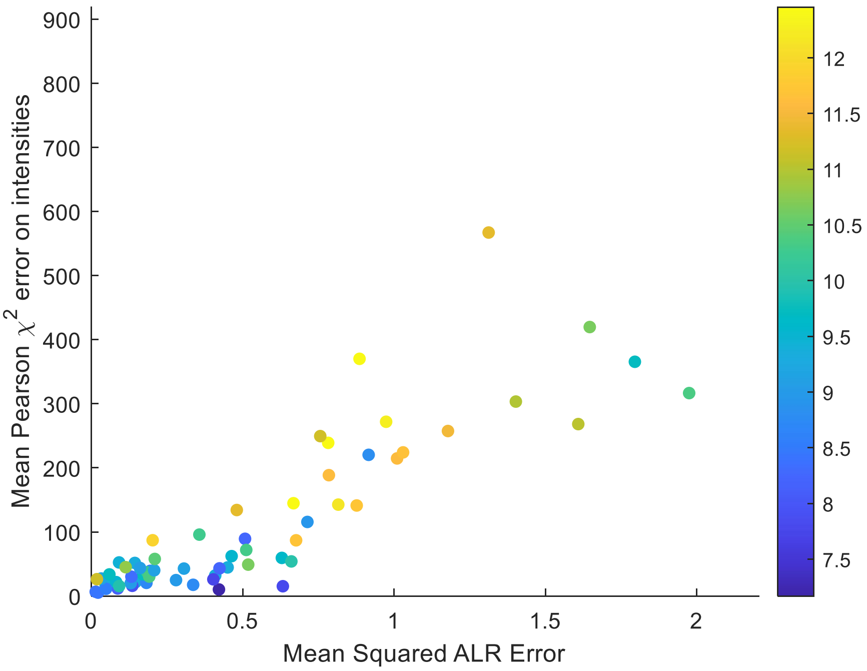

Figure S.15: Scatterplot of the mean Pearson χ^2 error on the probabilities (y-axis) versus the mean squared ALR error on the transformed isotope ratio's (x-axis). Each dot in the plot represents an isotope cluster. The colour represents the log10 AUC or sum intensity of the respective isotope cluster. It can be observed that a higher AUC generally leads to a higher error. Note that this plot includes 63 observations, i.e. 14 replicates of the DNA_SHORT2 compound in 5 charge states, minus 4 due to not finding the monoisotopic peak and minus 3 with an outlying MSE in ALR value of greater than 3.

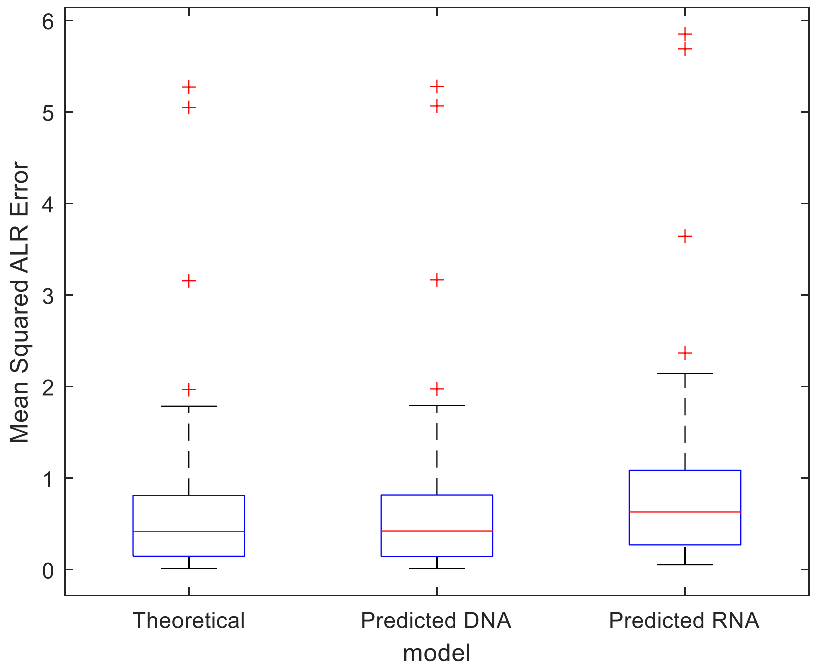

Figure S.16: Boxplot of the mean squared ALR error computed with the theoretical model (based on the elemental composition using BRAIN algorithm), predicted with the correct average DNA model and predicted using the misspecified average RNA model for compound DNA_SHORT2.

Supplementary materials for compound: RNA-like

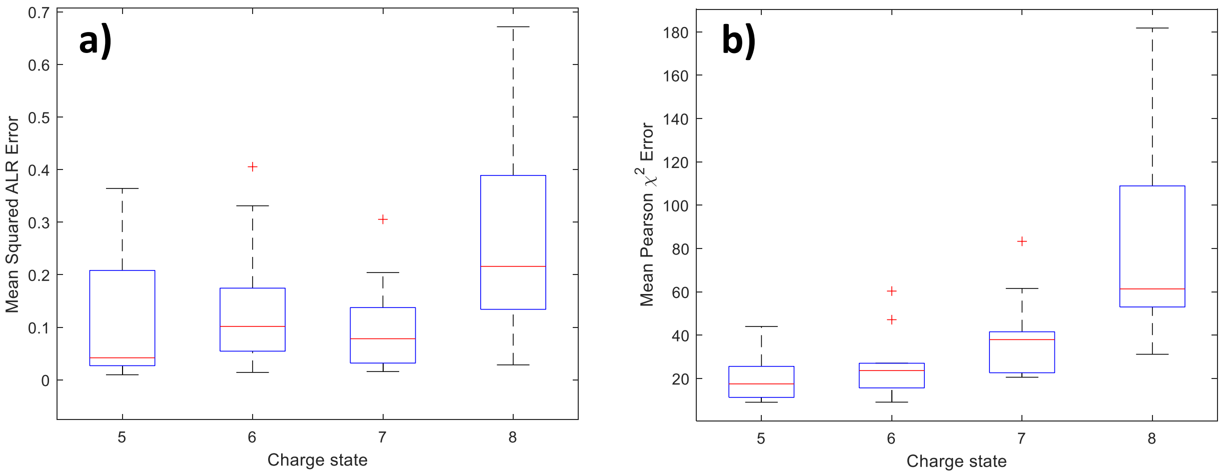

Figure S.17: The error distributions for compound RNA-like are provided as Tukey's box and whisker plots across the different charge states. Each box composes 12 repeated measurements of the compound over the LC-dimension of the experiment. Panel a) gives the distribution of mean squared ALR error. Panel b) gives the distribution of the mean Pearson's chi-squared error.

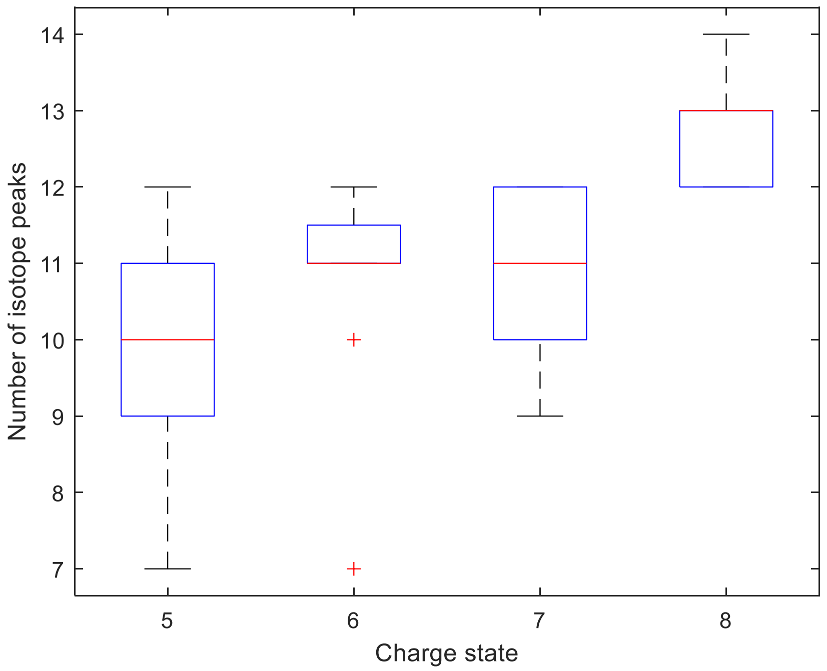

Figure S.18: Boxplots indicating the distribution of the number of observed isotope peaks in relation to the charge state. Each box composes 12 repeated measurements of the RNA-like compound over the LC-dimension of the experiment.

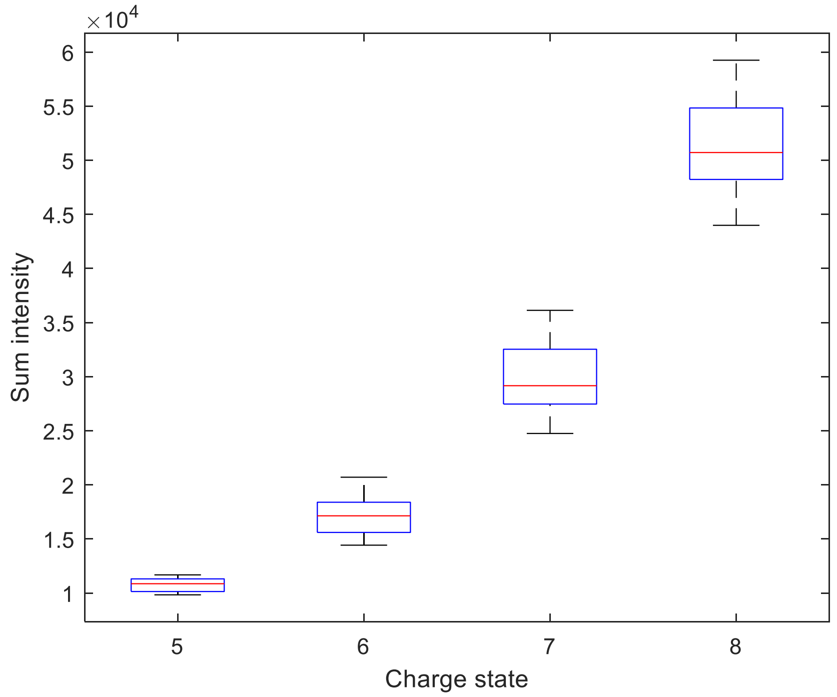

Figure S.19: Boxplots indicating the distribution of the AUC or sum intensity of an observed isotope pattern. Each box composes 12 repeated measurements of the RNA-like compound over the LC-dimension of the experiment.

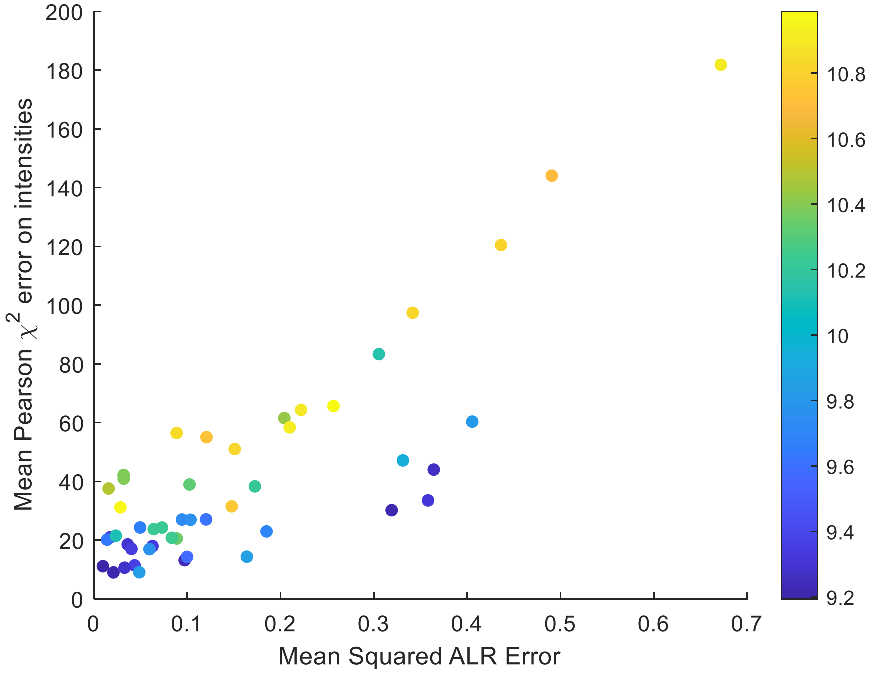

Figure S.20: Scatterplot of the mean Pearson χ^2 error on the probabilities (y-axis) versus the mean squared ALR error on the transformed isotope ratio's (x-axis). Each dot in the plot represents an isotope cluster. The colour represents the log10 AUC or sum intensity of the respective isotope cluster. It can be observed that a higher AUC generally leads to a higher error. Note that this plot includes 48 observations, i.e. 12 replicates of the RNA-like compound in 4 charge states.

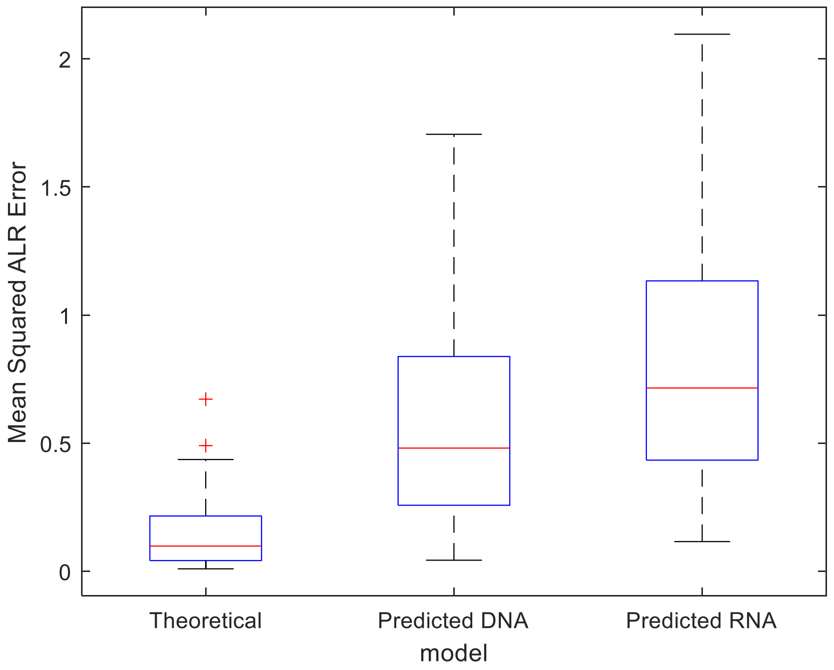

Figure S.21: Boxplot of the mean squared ALR error computed with the theoretical model (based on the elemental composition using BRAIN algorithm), predicted with the average DNA model and predicted using the average RNA model for compound RNA-like.

Supplementary materials for the software

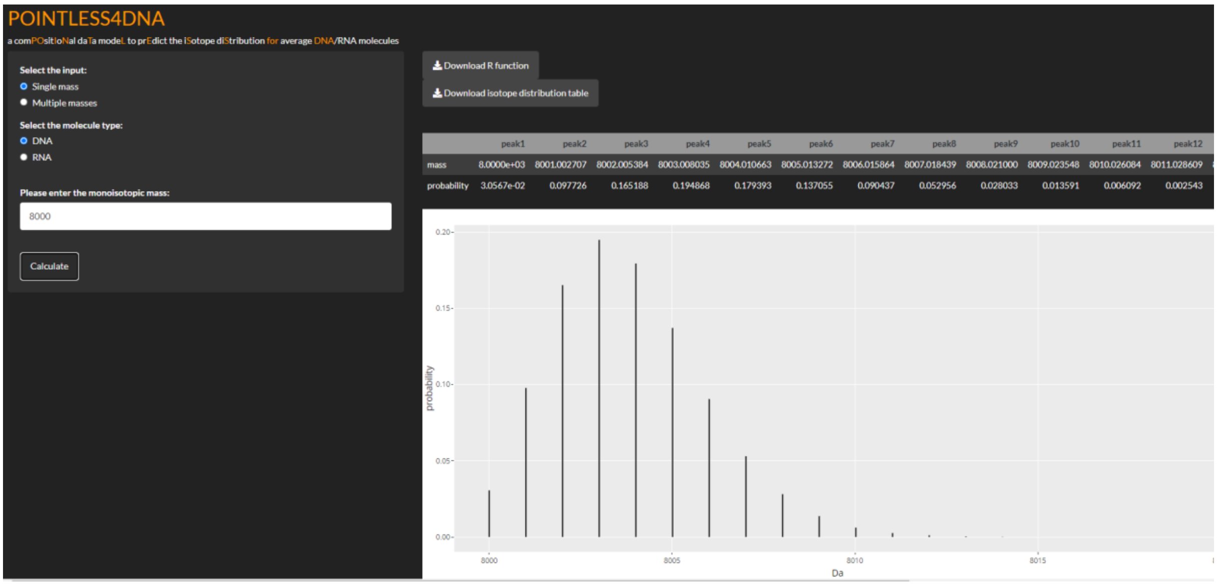

Figure S.22: The modelling framework for predicting the isotope distribution for average DNA/RNA molecules based on the compositional data model introduced in this manuscript has been made available to a wider public via an easy-to-use web application.
